# Physical Activity, Screen Time, and Academic Burden: A Cross-Sectional Analysis of Health among Chinese Adolescents

**DOI:** 10.3390/ijerph20064917

**Published:** 2023-03-10

**Authors:** Yiting E, Jianke Yang, Yifei Shen, Xiaojuan Quan

**Affiliations:** 1 Department of Sociology, Xi’an Jiaotong University, Xi’an 710049, China; 2 Department of Marxism, Xi’an Jiaotong University, Xi’an 710049, China

**Keywords:** physical activity, screen time, academic burden, adolescent health

## Abstract

This paper aims to analyze the effects of physical activity, screen time, and academic burden on adolescent health in China and compare their effects by using the nationally representative sample data from the CEPS (China Educational Panel Survey) cross-section data. This paper first uses regression analysis to examine the relationship between physical activity, screen time, academic burden and health among Chinese adolescents. Then, this paper uses the clustering analysis the influence of physical activity, screen time, and academic burden on the health of Chinese adolescents. The empirical results show that: (1) along with exercise, helping with the housework also has a clear health-promoting effect on adolescents; (2) the time spent surfing the Internet or playing video games, and heavy studying or homework off campus have a negative effect on adolescents’ self-rated health and mental health; (3) physical activity has the greatest impact on self-rated health, while screen time has the greatest impact on mental health, and academic burden is not the most important factor affecting adolescent health in China.

## 1. Introduction

The decline in adolescent health is a challenge common to many countries. According to the data released by UNICEF in 2020, more than 13 percent of adolescents aged 10–19 worldwide will have a mental illness [1]. Therefore, how to promote adolescent health has become a major concern for policy makers, educators, and parents in many countries. Scholars have actively explored the decline in adolescent health [2,3], and reached a basic consensus that socio-environmental factors, rather than genetic (biological) factors, are at play. For example, some scholars discovered that socio-environmental factors are particularly important in a period of dramatic lifestyle changes and increasing risks to adolescent health levels [4]. Although a number of studies have examined the effects of physical activity, screen time, and academic burden on adolescent health [5,6,7], few studies have been conducted on Chinese samples. The present study aims to fill this research gap by simultaneously considering the relationship of physical activity, screen time, and academic burden on health, and further examining different influences of the above three on health among Chinese adolescents.

## 2. Literature Review

### 2.1. Physical Activity, Screen Time and Academic Burden

#### 2.1.1. Physical Activity

Physical activity is one important health behavior and is defined as any bodily movement that is coordinated by skeletal muscle, resulting in increased energy expenditure above that of basal metabolism [8,9]. There is a large amount of research focusing on the linkage between physical activity and health. The Harvard Alumni Health Study began to focus on the social group differences in physical activity and the internal relationship between physical activity time, intensity, and health after discovering the effect of physical activity on life expectancy and the risk of chronic disease [10]. These studies greatly changed the public perception of physical activity by revealing that physical activities could play an important role in disease prevention and health promotion [11]. However, a global review showed that 81% of adults do not meet the WHO recommendation of at least 60 min of moderate to vigorous physical activity (MVPA) per day [12]. This may be explained by the fact that low and middle-income countries are now experiencing increased rates of inadequate physical activity levels.

Physical activity of adolescents is a fundamental determinant of a positive outlook on life [13], and engaging in more organized sports activities can foster lifelong attitudes and behaviors [14]. However, the lack of physical activity has not been effectively addressed, and the situation of young people is even more serious. According to a World Health Organization-funded study with a sample size of 1.6 million students between the ages of 11 and 17 in 146 countries/regions, 77.6% of boys and 84.7% of girls do not meet the recommended levels of physical activity [12]. Only 15% and 18.4% of children and adolescents in Europe and the United States, respectively, meet the WHO-recommended standard of “moderate to high-intensity physical activity for 60 min a day” [15,16]. The lack of physical activity levels is not only observed in the developed economies, but also in the low and- middle income countries as well. In China, only 29.9% of Chinese children and adolescents met the recommended criteria [17]. As a result, the risk of developing diseases such as obesity, hypertension, diabetes, cancer, etc., is greatly increased, and their present and future health is severely compromised [11]. Insufficient physical activity has become one of the most significant public health issues of this century, if not the most significant.

#### 2.1.2. Screen Time

As a modern health risk factor, sedentary behavior refers to sitting, reclining, or lying positions with energy expenditure ≤ 1.5 METs in the awake state [18]. Unlike physical activity, sedentary has caught attention in recent years [19,20]. However, it has similarities to physical inactivity in terms of prevalence and harmfulness. Sedentary behavior can be caused by screen time and academic burden in adolescence, although they have different origins. The former is mainly due to the addiction to the Internet and new media technologies, while the latter is caused by competitive education. 

It is generally acknowledged that physical activity declines while sedentary behavior increases from childhood to adolescence [21,22,23,24]. Additionally, with the rise of the mobile internet and new media technology, tablets and mobile phones have become a particularly integral part of adolescents’ daily lives. They frequently engage in sedentary screen-based activities, rather than outdoor exercise, sports, or play. They are strongly advised to limit their time spent sitting down, especially when engaging in recreational screen time [25]. Moreover, the sedentary behavior is device-specific. One cross-nation survey found the amount of time adolescents spent on computer and smart phone use increased dramatically while the time in watching television decreased slightly [26]. More studies show that screen time can deteriorate both physical and mental health. On the one hand, exposure to screens can increase adolescents’ risk of obesity [27]. On the other hand, excessive screen time might cause mental issues. Adolescents addicted to the Internet or video games and watch too much television are more likely to develop depression and various behavioral problems [28,29,30,31].

#### 2.1.3. Academic Burden

Academic burden can reduce sleep time and, therefore, has a significant negative impact on adolescents’ physical and mental health [32]. In addition, urban middle-class parents typically have high expectations for their children’s education and invest a variety of high-quality resources in studying so that their children can participate in shadow education. According to a survey of Ho Chi Minh City schools, 49% of parents believe that their children’s participation in shadow education adversely impacts their physical and mental health [33]. Such extracurricular tutoring can deprive adolescents of leisure time and social contact, thus increasing their tendency to develop internalized behavioral disorders such as anxiety and depression [34,35,36].

Chinese adolescents are exposed to intense academic competition and pressure and carry heavier academic burdens compared to those in other countries and regions. They frequently experience stress and anxiety regarding homework and after-school studies [37]. Although the government has designated the policy of reducing the academic burden on primary and secondary school students as one of the main responsibilities of compulsory education, it emphasizes that the government, schools, families, and society must cooperate in implementing the policy throughout the educational process and promote the healthy growth of students. However, the situation is still unfavorable. Chinese adolescents still carry a heavy academic burden [38].

### 2.2. Current Study

While exploring the social environmental factors affecting adolescent health in China and western countries, prior work has primarily examined the linkage between physical activity and health [39,40,41,42], or the effects of academic burden on physical health and mental health [43,44,45,46], and paid little attention to consider these three factors in an analytical framework. Our study of this issue not only provides a more comprehensive understanding of how these three factors are associated with adolescent health, but also helps develop targeted interventions and recommendations to parents and schools.

The main purpose of this study is to explore the relationships between physical activity, screen time, academic burden, and adolescent health in the Chinese context. Based on the previous literature review, two main research questions are proposed here. First, do physical activity, screen time and academic burden have significant effects on adolescent health? Second, which has the strongest effects on adolescent health among these three factors?

## 3. Methodology

### 3.1. Data

This study utilizes data from the China Educational Panel Survey (CEPS) conducted by National Survey Research Center (NSRC) at Renmin University of China. Taking the 2013–2014 data as the baseline, the CEPS applies a stratified, multistage sampling design with probability proportional to size (PPS), randomly selecting a school-based, nationally representative sample of approximately 20,000 students in 438 classrooms of 112 schools in 28 county-level units in mainland China. In the academic year 2014–2015, a follow-up visit was conducted with the eighth-grade students (seventh graders at the baseline survey) who participated in the baseline survey. The research object consists of 9449 students who were successfully followed up. By deleting the samples with missing values for the key variables, 8213 effective cases were finally obtained.

### 3.2. Measures

#### 3.2.1. Self-Rated Health and Mental Health

The CEPS questionnaire uses a five-point Likert scale to measure self-rated health. Respondents are required to answer the question “How is your overall health now”, and the corresponding options were “1. very bad, 2. not good, 3. fair, 4. better, 5. very good”. Since the number of cases in option 1 was too small, we combined it with option 2 to form an ordered variable with four categories: “1. not good, 2. average, 3. better, 4. very good”.

For mental health, we used a scale from the CEPS consisting of 10 questions measuring anxiety or depression. Each question corresponds to five options “1. never, 2. rarely, 3. sometimes, 4. often, 5. always”. The respondents were assigned a value of 1 for “3. sometimes, 4. often, 5. always” and a value of 0 for “1. never, 2. seldom.” The results of the new assignments for the 10 questions were then summed to obtain a count variable with values ranging from 0 to 10. The higher the number, the worse the mental health. 

#### 3.2.2. Physical Activity Time

Physical activity time includes daily physical exercise time and housework time. The average daily physical exercise time was calculated by replacing the value of each case that answers “more than 4 h” with 5 h. After that, it was operationalized into three categories: 1. less than or equal to quarter an hour, 2. more than quarter an hour less than or equal to half an hour, and 3. more than half an hour.

For housework time, this study replaced the case values over 4 h with 5 h and over 60 min with 60 min, thus calculating the average daily housework time, and then transformed it into three categories, “1. less than or equal to half an hour, 2. more than half an hour less than or equal to 1 h, 3. more than 1 h ”.

#### 3.2.3. Screen Time

Screen time was measured by the time spent in watching TV and surfing internet or playing game. The questionnaire firstly asked respondents how much time they watch TV per day, and the corresponding options were “1. none, 2. less than 1 h, 3. about 1–2 h, 4. about 2–3 h, 5. about 3–4 h, and 6. more than 4 h”. Next, the respondents were asked how much time they spent watching TV on weekends, and the corresponding options were “1. none, 2. less than 2 h, 3. about 2–4 h, 4. about 4–6 h, 5. about 6–8 h, and 6. more than 8 h”. We recoded values to each option with “1. 0 h, 2. 0.5 h, 3. 1.5 h, 4. 2.5 h, 5. 3.5 h, and 6. 4.5 h” and calculated the average daily TV watching time and converted it into three categories: 1. Less than or equal to 1 h, 2. More than 1 h less than or equal to 2 h, and 3. More than 2 h. Students were asked about the amount of time they spent online and playing games each day, and the corresponding options were set the same as those for “time spent watching TV each day. Following the above calculating way.

#### 3.2.4. Academic Burden Time

The homework time on campus and off campus per day was measured in the same way as the screen time, divided into “1. less than or equal to 2 h, 2. more than 2 h less than or equal to 4 h, 3. more than 4 h”.

#### 3.2.5. Control Variable

Social-economic characteristics include gender (female = 0; male = 1), household registration (agricultural household registration = 0, non-agricultural household registration = 1), years of parental education, family economic conditions (poor, medium, rich), whether they live with both parents (yes = 1, no = 0), only child status (yes = 1, no = 0), sleep time (1. less than and equal to 6 h; 2. more than 6 h and less than 8 h; 3. more than 8 h), and school residence status (yes = 1, no = 0). There were three survey questions applied to measure the extent of introversion. These three questions have four responses: 1. completely disagree, 2. not agree, 3. relatively agree, and 4. completely agree. By summing up option values for each question, a continuous variable with a range of 2 to 12 can be obtained. The greater the value, the more introverted the personality.

### 3.3. Data Analysis

Statistical analysis was performed using Stata 17.0. In accordance with the purpose of the study, three statistical methods are used in this paper. The first method is correlation analysis, which examines correlated relationship between physical activity, screen time, and academic burden. The second is a regression analysis in which we examine the effects of physical activity, screen time, and academic burden on adolescent health in the same framework. There are two dependent variables in this study: self-rated health and mental health. For self-rated health, it is an ordered variable, so we use the logistic regression model; for mental health, it is a counting variable, and its variance is greater than the mean value, which is an over-discrete case. Therefore, we use the negative binominal regression model in our study. The third analysis is the coefficient clustering analysis, which aims to compare the effects of physical activity and sedentary behavior on adolescent health, as well as the effect intensities of various types of physical activity and sedentary behavior. The clustering coefficients can classify the variables in the regression into distinct groups and assume that distinct groups of variables influence the dependent variable through a latent variable.

## 4. Results

### 4.1. Descriptive Statistic and Correlations

The results of the descriptive statistical analysis of relevant variables are displayed in Table 1. Most students felt that their overall health was generally poor. Only 29.93% of students believed their own health was good, 35.15% felt unwell, and 34.29% thought their health was generally good. The mean mental health score was 3.50, indicating that each student had an average of more than three types of anxiety or depression. The average daily exercise duration was 22 min, and nearly 50% of students exercise for less than 15 min per day. The average amount of time spent on housework was 1.08 h, but only 33.08% of students spent longer than 1 h. The average daily time spent watching television, browsing the Internet, and playing video games was 2.89 h, far exceeding the state-advocated 1 h limit. The average time spent on homework inside and outside of school was 2.95 h, which was about twice the national average (90 min).

In the sample used for analysis, the average number of years of education for parents is 11.09, indicating a junior high school education. 68.15% of the sampled population lived with both parents, while 44.69% of the students were only children. The proportions of the poor, the medium, and the wealthy families were 28.43%, 67.16%, and 4.41%, respectively. The percentages of students who slept less than or equal to 6 h, more than 6 h and less than or equal to 8 h, and more than 8 h were 5.83%, 51.13%, and 43.04%, respectively, and 30.37% of students lived in school from Monday to Thursday.

The results of the correlation analysis between physical activity, screen time, and aca demic burden are displayed in Table 2. The students spent more time on TV are doing more housework (r = 0.122). The time spent in using the Internet and playing video games was positively correlated with the time spent in exercising (r = 0.071) and TV watching (r = 0.395). The students who spent more time in homework on campus are more likely to engage in exercise (r = 0.064) and housework (r = 0.022). The correlation coefficient between homework time off campus is positively correlated with exercise time (r = 0.087) and study time in school (r = 0.907). As expected, students with more time on homework spent less time on TV watching (r = −0.021). 

### 4.2. Regression Analysis

The results of the regression analysis of physical activity, screen time, academic burden, and adolescent health are presented in Table 3. Overall health is the dependent variable in models (1) and (2), whereas mental health is the dependent variable in models (3) and (4). Models (1) and (3) only include control variables, while models (2) and (4) also consist of variables for physical activity, screen time, and academic burden. Household registration status or the number of siblings had no effects on self-rated health and mental health. The degree of students’ introverted personality has significantly lowered their self-rated health and increased their mental problems. Male students with higher family economic condition and longer sleep duration and those who lived with their parents showed a higher self-rated health score and were less likely to experienced mental health issues. Individual mental health has been significantly reduced by parents with a higher level of education, which has no bearing on the self-rated health status. Students who lived in the dorm had experienced more mental problems than those living off campus.

Model (2) indicates that students’ self-rated health will increase significantly when they engage in physical activities, no matter whether they are having exercising or doing housework. The cumulative probability of being classified in a certain health category is 13.6% and 45.8% lower for those with exercise time of 0.25–0.5 h; and those with exercise time of more than 0.5 h than those with exercise time of less than or equal to 0.25 h. In addition, the cumulative probability of being in a particular health category was 8.5% and 13.6% lower for those who worked for 0.5–1 h and more than 1 h than for those who worked for less than 0.5 h. As for screen time, watching TV seems to be beneficial to their self-rated health, while browsing the Internet, or playing video games seems to be opposite. Regarding the academic burden, students’ overall self-rated health is not significantly affected by the amount of time spent on homework in school. However, the cumulative probability of being in a particular health category was 25% higher for those who spent more than 4 h on schoolwork and homework than for those who spent less than or equal to 2 h.

Model (4) shows that those with exercise periods of 0.25–0.5 h and more than 0.5 h had lower mental problems by 4.6% and 8.5% compared to those with exercise duration of less than 0.25 h. However, the duration of housework does not impact mental health. In addition, those who watched more than 2 h of television per day had 7.1% more psychological problems than those who watched 1 h or less. Those who spent more time surfing the Internet and playing games had greater mental problems than those who spent less than 1 h. There was no significant effect on the mental health of the students who engaged in school study time every day. However, the mental health of the students who engaged in homework off campus for more than 4 h was significantly worse, which was 30.3% higher than that of the students who spent on homework off campus for less than 2 h.

### 4.3. Coefficient Clustering Analysis

The results of clustering analysis for the coefficients of physical activity, screen time, academic burden, and adolescent health are presented in Table 4. The comparison of self-rated health and mental health is calculated using models (2) and (4) from Table 4. As mentioned in the prior article, coefficient clustering analysis allows for the division of the variables in the regression into various groups and makes the assumption that these various groups of variables have an effect on the dependent variable through a latent variable. Here, we divide the independent variables into three groups: physical activity, screen time, and academic burden, and assume that these three groups have a coefficient-based effect on the dependent variable. After clustering, the coefficient (i.e., the total effect of a set of variables) is always a nonnegative number that represents only the magnitude of the influence and is independent of its direction.

It is clear that physical activity, screen time, and academic burden have a significant impact on adolescents’ physical and mental health, but to varying degrees. Physical activity has a greater impact on self-rated health than screen time (χ^2^ = 22.84,  p = 0.000) as well as academic burden (χ^2^ = 35.25, *p* = 0.000. However, screen time has a greater impact on mental health than physical activity and academic burden (χ^2^ = 16.69, *p* = 0.000). There is no difference between the impact intensity of physical activity and academic burden (χ^2^ = 1.23, *p* = 0.268).

## 5. Discussion

This study included physical activity, screen time, and academic burden in the same framework for comparative analysis and explored the independent effects of each factor on health. Previous research has provided a wealth of evidence for improving adolescent physical and mental health, suggesting that increased physical activity and reduced screen time contribute to adolescent health [47,48]. However, existing literature provides very limited insights into the academic burden, which is specific to China’s socio-cultural traditions and class mobility status. 

### 5.1. Main Findings

The first finding of this study is that the more the time spent exercising, the more the time spent on homework on campus and off campus. A strand of prior research has confirmed that academic burden was cited as the primary reason for not having sufficient physical activity during transition grades and high school years [49]; one possible explanation is that limited time necessitates the substitution of one behavior for another [50]. Therefore, homework or academic burden is the primary barrier for adolescents to engage in physical activity [51,52,53]. Regarding the social causes of the above results, this is attributed to differences in family social capital and parenting styles. The greater a family’s access to social resources, the greater the number of tasks children must master in and out of the classroom and the lengthier the learning period. For example, an empirical study in New Zealand found that while screen time type does appear to be implicated in academic achievement, the mechanism appears to be specific to higher socioeconomic status families [54]. 

Furthermore, the study showed that the time spent on the Internet and playing video games was positively correlated with the time spent in physical activity, and moderate screen time is good for physical and mental health [55]. Previous studies have revealed that the greater screen time will impairs cognition [56]. The research conducted by Zhang et al., supports our findings. He believes that screen-based activities can be properly implemented to meet cognitive development requirements. For young children, it appears essential to encourage physical activity and participation in organized and unorganized activities, as well as to adhere to screen time recommendations for cognitive development [42]. As the research results above, watching TV seems to be beneficial to adolescents’ self-rated health, while browsing the Internet, or playing video games seems to be opposite. One possible explanation is that Chinese students are believed to have high academic burden and pressure due to high expectations of their parents and fierce competitions with their peers [57], so they do not have much time to watch TV. Meanwhile, from a global perspective, adolescents’ internet addiction has become a worldwide problem, and it has high risks to physical and mental health [58,59,60]. However, with the extensive development of the Internet, the pooled Internet addiction detection rate of Chinese college students was 11%, which is higher than in some other countries [61]. Second, our study found that physical activity consisting of exercise and housework time has significant effects on adolescents’ self-rated health, while exercise time instead of housework time has negatively effects on mental health. In other words, the more exercise, the less likelihood of a mental problem. In terms of screen time, TV watching seemed to increase their self-rated health ranking. Compared to those watching less one hour per day, those watching TV more than two hours are more likely to have mental problem. As for those students spending more time in online surfing and game playing, they are more likely to low their self-rated health ranking and have more mental problems. During COVID-19, many people live a sedentary lifestyle, including lack of physical activity and long screen time, and have a poor psychological status, which may lead to considerable health risks [62,63]. We also found that time spent on schoolwork had no significant effects on self-rated health and mental health. However, compared to those students spending on study and homework off campus less 2 h, those who spent more than 4 h reduced their self-rate health ranking and increased their mental health problems. This result has important policy implications. It suggests that the school and family should work together and reduce the over burden of their students’ study on campus and homework off campus. In addition, schools should emphasize comprehensive wellness strategies to address multiple behaviors to maximize student health and academic success [64].

The final and most important finding is that the academic burden is not the most significant risk factor for Chinese adolescents’ health. In other words, physical activity has a greater effect on self-rated health than screen time and academic burden, while screen time has a greater impact on mental health than both physical activity and academic burden. One possible explanation could be that physical activity may improve functional status, and good function status has related to self-rated health status [65,66]. As for screen time, on the one hand, people who spend more time in front of the screen have more sleep problems, which may affect their mental state and lead to increased depression and anxiety [67,68]. On the other hand, adolescents’ internet addiction will cause loneliness and low self-esteem, which are harmful to mental health [69,70,71]. This indicates that a separate physical activity promotion policy does not necessarily lead to a reduction in screen time in practice. Nonetheless, school intervention is still required to increase students’ physical activity levels [72,73,74]. This finding provides new empirical evidence for managing screen time with an emphasis on increasing physical activity and reducing academic burden, as physical activity, screen time, and academic burden have different health effects. 

Furthermore, digital media have become an important way for individuals to access information, communication, and entertainment. In addition to positive government interventions, families and schools should give high priority to adolescents’ use of electronic devices, which not only contributes to children’s educational outcomes but also to their health status.

The current cross-sectional study addresses this research gap by examining the complex associations among physical activity, screen time, and academic burden and health. With the rapid growth in economy, smartphones and tablets are gaining widespread use among children and youth and may already be key contributors to screen time. In addition to positive government interventions, families and schools should give high priority to adolescents’ use of electronic devices, and well-designed trials that are currently accruing participants are examining innovative and more comprehensive strategies for reducing screen time [75]. In Finland, a specific content on physical activity and screen time based on Health Action Process Approach model was integrated into routinely scheduled three health education lessons with the help of educational material in secondary schools [76].

### 5.2. Study Limitations

There are, however, several limitations in this paper. First, the depth and accuracy of the data analysis are limited since the data used were not collected through a special survey, which makes it difficult to measure all relevant variables. An example of this would be the lack of information regarding the type, intensity, and content of physical activity when playing games and surfing the internet. Second, despite the facts that the analysis samples are nationally representative, they are all eighth-graders, or junior high school students. The research on the health and related behaviors of adolescents of other ages requires additional investigation and analysis. Third, this study is the cross-sectional character of our dataset. It is a consensus that results derived from a cross-sectional study cannot address the issue of causality, thus longitudinal studies are expected in order to explore the causal relationships.

## 6. Conclusions

On the basis of national survey data, this study examines the relationship among physical activity, screen time, and academic burden, and compares their effects on the physical and mental health of adolescents. The key findings are as follows: first, in addition to physical exercise, helping with housework also has a clear health-promoting effect on adolescents. Second, screen time and academic pressure will generally have a detrimental effect on adolescents’ health, particularly the time spent surfing the Internet, playing video games, excessive studying outside of school. Third, screen time has the greatest impact on mental health, while physical activity has the greatest impact on self-rated health. It appears that academic burden is not the greatest social risk factor for adolescents’ health in China. In addition, the current research results here are worthy of further study in the future to explore the influencing factors and mechanisms of adolescent health in China, in order to find a balance between all parties and provide more solid and reliable scientific basis for adolescent health promotion. This implies that policy makers should make different policy key points in term of how to improve adolescents’ self-rated health and mental health.

## Figures and Tables

**Table 1:** Descriptive results for variables.

Variables	Mean % (S.D.)		Category and Proportion
Dependent variable			
Self-rated health	\	\	1. very bad (6.37%), 2. not very good (28.78%), 3. Average (34.92%), 4. good (29.93%)
Mental health	3.50	3.21	
Independent variable			
Physical activity			
Exercise time/day·hour	0.37	0.41	1. ≤0.25 h (49.91%), 2. 0.25–0.5 h (30.88%), 3. >0.5 h (19.21%)
Housework time/day·hour	1.08	1.16	1. ≤0.5 h (39.08%), 2. 0.5–1 h (27.83%), 3. >1 h (33.08%)
Screen time			
TV watching time/day·hour	1.54	1.31	1. ≤1 h (43.30%), 2. 1–2 h (32.31%), 3. >2 h (24.39%)
Online and game time/day·hour	1.35	1.40	1. ≤1 h (52.68%), 2. 1–2 h (26.30%), 3. >2 h (21.02%)
Academic burden			
School study time/day·hour	1.71	0.95	1. ≤2 h (72.53%), 2. 2–4 h (25.16%), 3. >4 h (2.31%)
Off campus study and homework time/day·hour	1.24	1.74	1. ≤2 h (76.60%), 2. 2–4 h (16.33%), 3. >4 h (7.07%)
Control variable			
Gender			0. Female (48.12%), 1. Male (51.88%)
Registered residence			0. Agricultural household registration (54.02%), 1. Non agricultural household registration (45.98%)
Maximum education years of parents	11.09	3.10	\
Whether to live with both parents	\	\	0. No (31.85%), 1. Yes (68.15%)
Family economic conditions	\	\	1. Poor (28.43%), 2. Medium (67.16%), 3. Rich (4.41%)
Only child	\	\	0. No (55.31%), 1. Yes (44.69%)
Degree of introversion (personality)	5.86	2.15	\
Sleep time	\	\	1. ≤6 h (5.83%), 2. 6–8 h (51.13%), 3. >8 h (43.04%)
School residence status	\	\	0. No (69.63%), 1. Yes (30.37%)

**Table 2:** Correlation analysis of physical activity, screen time, and academic burden.

Variable	1	2	3	4	5	6
Physical activity						
1. Exercise time/day	1.000					
2. Housework time/day	−0.014	1.000				
Screen time						
3. TV watching time/day	−0.005	0.122 ***	1.000			
4. Online and game time/day	0.071 ***	−0.015	0.395 ***	1.000		
Academic burden						
5. School study time/day	0.064 ***	0.022 **	−0.003	0.011	1.000	
6. Off campus study and homework time/day	0.087 ***	−0.015	−0.021 *	0.010	0.907 ***	1.000

Note: *** *p* < 0.01, ** *p* < 0.05, * *p* < 0.1.

**Table 3:** Regression analysis of physical activity, screen time, academic burden, and adolescent health.

Variables	Overall Health		Mental Health	
	(1)	(2)	(3)	(4)
Control variable				
Gender (reference group: female)	0.236 ***	0.187 ***	−0.029	−0.047 **
Household registration (reference group: agricultural household registration)	0.012	0.010	0.022	0.017
Degree of introversion	−0.166 ***	−0.161 ***	0.113 ***	0.114 ***
Maximum education years of parents	0.009	0.007	−0.013 ***	−0.010 **
Family economic conditions (reference group: poor)				
secondary	0.248 ***	0.265 ***	−0.070 ***	−0.079 ***
affluent	0.689 ***	0.728 ***	−0.121 **	−0.157 ***
Only child (reference group: no)	0.071	0.068	−0.034	−0.030
Whether to live with both parents (reference group: no)	0.210 ***	0.208 ***	−0.073 ***	−0.059 **
Residential (reference group: no)	−0.133 **	−0.100	0.125 ***	0.134 ***
Daily sleep time (reference group: ≤6 h)				
6–8 h	0.374 ***	0.367 ***	−0.246 ***	−0.205 ***
>8 h	0.594 ***	0.578 ***	−0.409 ***	−0.372 ***
Physical activity				
Exercise time/day (reference group: ≤0.25 h)				
0.25–0.5 h		0.146 ***		−0.047 *
>0.5 h		0.612 ***		−0.089 ***
Housework time/day (reference group: ≤0.5 h)				
0.5–1 h		0.089 *		−0.029
>1 h		0.146 ***		0.037
Screen time				
TV watching time/day (reference group: ≤1 h)				
1–2 h		0.098 **		0.023
>2 h		0.168 ***		0.069 **
Online and game time/day (reference group: ≤1 h)				
1–2 h		−0.117 **		0.061 **
>2 h		−0.206 ***		0.260 ***
Academic burden				
School study time/day (reference group: ≤2 h)				
2–4 h		0.104		−0.038
>4 h		0.008		−0.142
Off campus study and homework time/day (reference group: ≤2 h)				
2–4 h		−0.113		0.062
>4 h		−0.223 **		0.265 ***
cut1	−2.783 ***	−2.579 ***		
cut2	−0.618 ***	−0.399 **		
cut3	0.949 ***	1.189 ***		
Intercept			1.065 ***	0.901 ***
Number of groups	112	112	112	112
Log likelihood	−10,054.65	−9985.75	−19,234.35	−19,167.19

Note: due to the limitation of length, only the fixed effect analysis results are listed. In addition, the corresponding standard error is not provided, and those who require it should contact the author. *** *p* < 0.01, ** *p* < 0.05, * *p* < 0.1.

**Table 4:** The results of cluster analysis of physical activity, screen time, academic burden, and adolescent health.

Variable	Self-Rated Health	Mental Health
Physical activity	0.234 ***	0.045 ***
Screen time	0.088 ***	0.114 ***
Academic burden	0.055 ***	0.054 ***

Note: *** *p* < 0.01.

## Data Availability

Publicly available datasets were analyzed in this study. These data can be found here: http://ceps.ruc.edu.cn/, accessed on 24 February 2023.

## References

[1] Keeley B. (2021). The State of the World’s Children 2021: On My Mind—Promoting, Protecting and Caring for Children’s Mental Health.

[2] Coady S.A., Jaquish C.E., Fabsitz R.R., Larson M.G., Cupples L.A., Myers R.H. (2002). Genetic variability of adult body mass index: A longitudinal assessment in Framingham families. Obes. Res..

[3] Dolgin E. (2015). The myopia boom. Nature.

[4] Zhou M., Ding X. (2021). Internet use, depression, and cognitive outcomes among Chinese adolescents. J. Community Psychol..

[5] Joseph D.M., Alivia R.N., Guy F., Diane G.J., Thomas L.S., Allison P.K. (2022). The association of physical activity, sleep, and screen time with mental health in Canadian adolescents during the COVID-19 pandemic: A longitudinal isotemporal substitution analysis. Ment. Health Phys. Act..

[6] Tomoka K., Takeo F., Satomi D., Yui Y. (2022). Association between Hope for the Future and Academic Performance in Adolescents: Results from the K-CHILD Study. Int. J. Environ. Res. Public Health.

[7] Waldman D.Z., Kristie R. (2022). Physical Activity, Sports Participation, and Psychosocial Health in Adolescents with Hearing Loss. J. Adolesc. Health.

[8] Qin W., Guo Y. (2023). Physical activity among older African Americans: The role of diabetes diagnosis and mastery. J. Ethn. Cult. Divers. Soc. Work.

[9] Shakoor H., Platat C., Ali H.I., Ismail L.C., Al Dhaheri A.S., Bosevski M., Apostolopoulos V., Stojanovska L. (2023). The benefits of physical activity in middle-aged individuals for cardiovascular disease outcomes. Maturitas.

[10] Raven P.B., Wasserman D.H., Squires W.G., Murray T.D. (2012). Exercise Physiology.

[11] Blair S.N. (2009). Physical inactivity: The biggest public health problem of the 21st century. Br. J. Sport. Med..

[12] Guthold R., Stevens G.A., Riley L.M., Bull F.C. (2020). Global trends in insufficient physical activity among adolescents: A pooled analysis of 298 population-based surveys with 1·6 million participants. Lancet Child Adolesc. Health.

[13] Malinowska-Cieślik M., Mazur J., Nałęcz H., Małkowska-Szkutnik A. (2019). Social and behavioral predictors of adolescents’ positive attitude towards life and self. Int. J. Environ. Res. Public Health.

[14] Emm-Collison L.G., Lewis S., Reid T., Matthews J., Sebire S.J., Thompson J.L., Salway R., Jago R. (2019). Striking a balance: Physical activity, screen-viewing and homework during the transition to secondary school. Int. J. Environ. Res. Public Health.

[15] Currie C., Zanotti C., Morgan A., Currie D., de Looze M., Roberts C., Samdal O., Smith O.R., Barnekow V. (2012). Social Determinants of Health and Well-Being among Young People: Health Behaviour in School-Aged Children (HBSC) Study: International Report from the 2009/2010 Survey.

[16] Kann L., McManus T., Harris W.A., Shanklin S.L., Flint K.H., Queen B., Lowry R., Chyen D., Whittle L., Thornton J. (2018). Youth risk behavior surveillance—United States, 2017. MMWR Surveill. Summ..

[17] Fan X., Cao Z.-B. (2017). Physical activity among Chinese school-aged children: National prevalence estimates from the 2016 Physical Activity and Fitness in China—The Youth Study. J. Sport Health Sci..

[18] Tremblay M.S., Aubert S., Barnes J.D., Saunders T.J., Carson V., Latimer-Cheung A.E., Chastin S.F., Altenburg T.M., Chinapaw M.J. (2017). Sedentary behavior research network (SBRN)–terminology consensus project process and outcome. Int. J. Behav. Nutr. Phys. Act..

[19] Iannotti R.J., Wang J. (2013). Trends in physical activity, sedentary behavior, diet, and BMI among US adolescents, 2001–2009. Pediatrics.

[20] Matthews C.E., Chen K.Y., Freedson P.S., Buchowski M.S., Beech B.M., Pate R.R., Troiano R.P. (2008). Amount of time spent in sedentary behaviors in the United States, 2003–2004. Am. J. Epidemiol..

[21] Arundell L., Fletcher E., Salmon J., Veitch J., Hinkley T. (2016). A systematic review of the prevalence of sedentary behavior during the after-school period among children aged 5–18 years. Int. J. Behav. Nutr. Phys. Act..

[22] Corder K., van Sluijs E.M., Ekelund U., Jones A.P., Griffin S.J. (2010). Changes in children’s physical activity over 12 months: Longitudinal results from the SPEEDY study. Pediatrics.

[23] Dumith S.C., Gigante D.P., Domingues M.R., Kohl III H.W. (2011). Physical activity change during adolescence: A systematic review and a pooled analysis. Int. J. Epidemiol..

[24] Marks J., Barnett L.M., Strugnell C., Allender S. (2015). Changing from primary to secondary school highlights opportunities for school environment interventions aiming to increase physical activity and reduce sedentary behaviour: A longitudinal cohort study. Int. J. Behav. Nutr. Phys. Act..

[25] Bull F.C., Al-Ansari S.S., Biddle S., Borodulin K., Buman M.P., Cardon G., Carty C., Chaput J.-P., Chastin S., Chou R. (2020). World Health Organization 2020 guidelines on physical activity and sedentary behaviour. Br. J. Sport. Med..

[26] Bucksch J., Sigmundova D., Hamrik Z., Troped P.J., Melkevik O., Ahluwalia N., Borraccino A., Tynjälä J., Kalman M., Inchley J. (2016). International trends in adolescent screen-time behaviors from 2002 to 2010. J. Adolesc. Health.

[27] Rocka A., Jasielska F., Madras D., Krawiec P., Pac-Kożuchowska E. (2022). The impact of digital screen time on dietary habits and physical activity in children and adolescents. Nutrients.

[28] Gentile D.A., Choo H., Liau A., Sim T., Li D., Fung D., Khoo A. (2011). Pathological video game use among youths: A two-year longitudinal study. Pediatrics.

[29] Hamer M., Yates T., Sherar L.B., Clemes S.A., Shankar A. (2016). Association of after school sedentary behaviour in adolescence with mental wellbeing in adulthood. Prev. Med..

[30] Kleppang A.L., Steigen A., Finbråten H. (2019). The association between screen time and psychological distress in Norwegian adolescents. Eur. J. Public Health.

[31] Robinson T.N., Banda J.A., Hale L., Lu A.S., Fleming-Milici F., Calvert S.L., Wartella E. (2017). Screen media exposure and obesity in children and adolescents. Pediatrics.

[32] Lee M., Larson R. (2000). The Korean ‘examination hell’: Long hours of studying, distress, and depression. J. Youth Adolesc..

[33] Dang H.-A. (2011). A bird’s-eye view of the private tutoring phenomenon in Vietnam. IIAS Newsl..

[34] Kuan P.-Y. (2018). Effects of cram schooling on academic achievement and mental health of junior high students in Taiwan. Chin. Sociol. Rev..

[35] Mahmud R. (2021). Learning in the shadows: Parents’ investments, family burden, and students’ workload in Dhaka, Bangladesh. Asia Pac. Educ. Rev..

[36] Tan J. (2012). Confronting the Shadow Education System: What Government Policies for What Private Tutoring?.

[37] Hong E., Mason E., Peng Y., Lee N. (2015). Effects of homework motivation and worry anxiety on homework achievement in mathematics and English. Educ. Res. Eval..

[38] Zhu X., Haegele J.A., Tang Y., Wu X. (2017). Physical Activity and Sedentary Behaviors of Urban Chinese Children: Grade Level Prevalence and Academic Burden Associations. BioMed Res. Int..

[39] Balaji S., Karthik R., Durga R., Harinie S., Ezhilvanan M. (2018). Intensity of physical activity among school going adolescents in Chennai, South India. Int. J. Community Med. Public Health.

[40] Marconnot R., Marín-Rojas A.L., Delfa-de-la-Morena J.M., Pérez-Corrales J., Gueita-Rodríguez J., Fernández-de-Las-Peñas C., Palacios-Ceña D. (2019). Recognition of barriers to physical activity promotion in immigrant children in Spain: A qualitative case study. Int. J. Environ. Res. Public Health.

[41] Nguyen N.-M., Dibley M.J., Tang H.K., Alam A. (2017). Perceptions and practices related to obesity in adolescent students and their programmatic implications: Qualitative evidence from Ho Chi Minh City, Vietnam. Matern. Child Health J..

[42] Zhang Z., Wiebe S.A., Rahman A.A., Carson V. (2022). Longitudinal associations of subjectively-measured physical activity and screen time with cognitive development in young children. Ment. Health Phys. Act..

[43] Kim K.M., Kim D., Chung U.S. (2020). Investigation of the trend in adolescent mental health and its related social factors: A multi-year cross-sectional study for 13 years. Int. J. Environ. Res. Public Health.

[44] Kyeong L.W. (2013). Self-compassion as a moderator of the relationship between academic burn-out and psychological health in Korean cyber university students. Personal. Individ. Differ..

[45] Zhang D., Hong J., Chen S., Liu Y. (2022). Associations of physical activity with academic achievement and academic burden in Chinese children and adolescents: Do gender and school grade matter?. BMC Public Health.

[46] Zhu X., Haegele J.A., Liu H., Yu F. (2021). Academic stress, physical activity, sleep, and mental health among Chinese adolescents. Int. J. Environ. Res. Public Health.

[47] Babic M., Colyvas K., Morgan P., Plotnikoff R., Lonsdale C., Lubans D. (2017). Longitudinal associations between recreational screen-time and mental health in Australian adolescents: A cross-lagged panel analysis. J. Sci. Med. Sport.

[48] Hasan M.K.C., Abdullah F., Firdaus M.K.Z.H., Jamaludin F.I.C. (2021). Does physical activity and body weight status determine musculoskeletal health among adolescents in Malaysia?. Enfermería Clínica.

[49] Pearson N., Braithwaite R., Biddle S.J., van Sluijs E.M., Atkin A.J. (2014). Associations between sedentary behaviour and physical activity in children and adolescents: A meta-analysis. Obes. Rev..

[50] Gilchrist J.D., Battista K., Patte K.A., Faulkner G., Carson V., Leatherdale S.T. (2021). Effects of reallocating physical activity, sedentary behaviors, and sleep on mental health in adolescents. Ment. Health Phys. Act..

[51] Knowles A.-M., Niven A., Fawkner S. (2011). A qualitative examination of factors related to the decrease in physical activity behavior in adolescent girls during the transition from primary to secondary school. J. Phys. Act. Health.

[52] Mulvihill C., Rivers K., Aggleton P. (2000). Views of young people towards physical activity: Determinants and barriers to involvement. Health Educ..

[53] Trost S.G., Pate R.R., Saunders R., Ward D.S., Dowda M., Felton G. (1997). A prospective study of the determinants of physical activity in rural fifth-grade children. Prev. Med..

[54] Skvarc D.R., Penny A., Harries T., Wilson C., Joshua N., Byrne L.K. (2021). Type of screen time and academic achievement in children from Australia and New Zealand: Interactions with socioeconomic status. J. Child. Media.

[55] Scarabottolo C.C., Tebar W.R., Araújo Guerra P.H., Martins C.M.d.L., Ferrari G., Beretta V.S., Christofaro D.G.D. (2022). Association between Different Domains of Sedentary Behavior and Health-Related Quality of Life in Adults: A Longitudinal Study. Int. J. Environ. Res. Public Health.

[56] Donald R.J., Enid S.R.M., Joan V.A., Esteban B.E. (2022). The convergent effects of primary school physical activity, sleep, and recreational screen time on cognition and academic performance in grade 9. Front. Hum. Neurosci..

[57] Sun J., Dunne M.P., Hou X.-Y. (2012). Academic stress among adolescents in China. Australas. Epidemiol..

[58] Abebe Z.E., Tadesse T., Abebaw T.S., Mamuye A.M., Yideg Y.G., Tadele A.F., Walle A.G., Jemere A.T., Chekol A.E., Tilahun M.Z. (2022). Internet Addiction and Its Associated Factors Among African High School and University Students: Systematic Review and Meta-Analysis. Front. Psychol..

[59] Gavurova B., Ivankova V., Rigelsky M., Mudarri T. (2022). Internet addiction in socio-demographic, academic, and psychological profile of college students during the COVID-19 pandemic in the Czech Republic and Slovakia. Front. Public Health.

[60] Imen M., Arij N., Imen S., Ines B., Sana E.M. (2023). Adverse childhood experiences and sleep disorders among Tunisian adolescents: The mediating role of internet addiction. Child Abus. Negl..

[61] Shao Y., Zheng T., Wang Y., Liu L., Chen Y., Yao Y. (2018). Internet addiction detection rate among college students in the People’s Republic of China: A meta-analysis. Child Adolesc. Psychiatry Ment. Health.

[62] Akulwar-Tajane I., Shah A.V., Naik P.H., Parmar K.K. (2021). Rethinking Screen Time during COVID-19: Impact on Sleep and Academic Performance in Physiotherapy Students. Health Res..

[63] Qin F., Song Y., Nassis G.P., Zhao L., Dong Y., Zhao C., Feng Y., Zhao J. (2020). Physical Activity, Screen Time, and Emotional Well-Being during the 2019 Novel Coronavirus Outbreak in China. Int. J. Environ. Res. Public Health.

[64] Howie E.K., Joosten J., Harris C.J., Straker L.M. (2020). Associations between meeting sleep, physical activity or screen time behaviour guidelines and academic performance in Australian school children. BMC Public Health.

[65] Chen J.D. (1995). Benefits of physical activity on nutrition and health status: Studies in China. Asia Pac. J. Clin. Nutr..

[66] Rami R.K.K., Krishna R.B., Papa R.A. (2004). Interaction among body composition, self-rated health and functional status of the elderly in an Indian population. Asia Pac. J. Clin. Nutr..

[67] Cyl A., Rt B. (2021). Sleep duration does not mediate the association between screen time and adolescent depression and anxiety: Findings from the 2018 National Survey of Children’s Health. Sleep Med..

[68] Panchali M., Jagmeet M. (2022). Impact of screen time during COVID-19 on eating habits, physical activity, sleep, and depression symptoms: A cross-sectional study in Indian adolescents. PLoS ONE.

[69] Barton E.H., Dimitrios V., Ralph N., Akosua T., Kwame B.P., Charles O. (2022). The Impact of Mental Health Predictors of Internet Addiction among Pre-Service Teachers in Ghana. Behav. Sci..

[70] Mohsen K., Masumeh K., Aida F. (2022). Personality traits and college students’ internet addiction: The mediating roles of general health and self-esteem. Scand. J. Psychol..

[71] Mwakilama E.P., Jamu E.S., Senganimalunje L., Manda T.D. (2022). Data from “Internet Addiction and Mental Health among College Students in Malawi”. J. Open Psychol. Data.

[72] Ahmed K.R., Kolbe-Alexander T., Khan A. (2022). Effectiveness of a school-based intervention on physical activity and screen time among adolescents. J. Sci. Med. Sport.

[73] Gråstén A. (2017). School-based physical activity interventions for children and youth: Keys for success. J. Sport Health Sci..

[74] Watson A., Timperio A., Brown H., Best K., Hesketh K.D. (2017). Effect of classroom-based physical activity interventions on academic and physical activity outcomes: A systematic review and meta-analysis. Int. J. Behav. Nutr. Phys. Act..

[75] Saunders T.J., Vallance J.K. (2017). Screen Time and Health Indicators among Children and Youth: Current Evidence, Limitations and Future Directions. Appl. Health Econ. Health Policy.

[76] Anne-Mari J., Tommi V., Olavi P., Harri S., Kari T., Henri V.-Y., Anna B., Minna A. (2015). KIDS OUT! Protocol of a brief school-based intervention to promote physical activity and to reduce screen time in a sub-cohort of Finnish eighth graders. BMC Public Health.

